# Usefulness of a Cannula with a Flexible Tip (Swing Tip) for Managing Severe Biliary Stricture

**DOI:** 10.1155/2018/7125714

**Published:** 2018-12-12

**Authors:** Daisuke Uchida, Hironari Kato, Yosuke Saragai, Saimon Takada, Shinichiro Muro, Takeshi Tomoda, Kazuyuki Matsumoto, Shigeru Horiguchi, Hiroyuki Okada

**Affiliations:** Department of Gastroenterology, Okayama University Hospital, Okayama, Japan

## Abstract

**Background and Aims:**

Biliary stenting for the treatment of biliary stricture is the most common indication of ERCP, but the procedure is sometimes unsuccessful because of severe strictures. The Swing Tip is useful for passing through severe strictures because it has a manually operable tip. The efficacy of using a Swing Tip was retrospectively evaluated.

**Methods:**

The 2353 patients who underwent ERCP for biliary stenting at our facility between January 2012 and July 2018 were enrolled. In all patients, procedures were begun using tapered tip-catheters, and Swing Tips were used if the procedure was found to be difficult with other devices. The indication for switching to the Swing Tip and the technical success rate were retrospectively evaluated.

**Results:**

A total of 99 patients (4.2%) underwent ERCP using the Swing Tip, including 49 patients for the selection of biliary branches and 50 for exchanging guidewires for rigid ones. In these patients, biliary stenting was successful in 22 patients (44.9%) and 45 patients (90%), respectively. The other 32 patients with failed endoscopic biliary drainage were treated via alternative approaches, such as percutaneous procedures, surgeries, or conservative treatments. There were no adverse events associated with the Swing Tip.

**Conclusion:**

The Swing Tip was technically feasible especially for exchanging guidewires during ERCP. Percutaneous procedures or surgical treatments can be avoided by using the Swing Tip.

**Ethical Statements:**

This study was approved by the institutional review board of Okayama University. All subjects provided informed consent. The study was registered in the UMIN protocol registration system (identification number UMIN 000033692).

## 1. Introduction

Endoscopic retrograde cholangiopancreatography (ERCP) is a very important procedure for the treatment of pancreatobiliary disease, and its approaches have become multifaceted in recent years. The primary challenge during ERCP is selective cannulation. While various devices and methods for selective cannulations have been developed [1–5], the difficulty of the ERCP procedure is due to more than just the selective cannulation of papilla.

**Figure 1:**
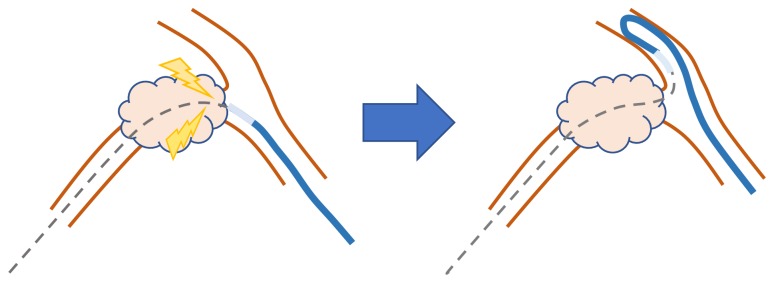
It is sometimes difficult to lead a catheter through a stricture in the corner of the bile duct.

Selecting a targeted biliary branch is sometimes difficult even if flexible guidewires are used because of the complicated anatomies of some crooked bile ducts. Furthermore, even when a guidewire is advanced, devices may not be able to be led over the guidewire (Figure 1). The Swing Tip was developed as a cannula with a flexible tip and is compatible with a 0.035-inch guidewire. Igarashi et al. reported the efficacy of the Swing Tip for selective cannulation during ERCP [6]. The characteristics of this cannula may help resolve other technical problems described above.

In the present study, we evaluated the efficacy of the Swing Tip for resolving technical issues encountered during ERCP without papillary cannulation.

## 2. Methods

We retrospectively investigated 2353 patients who had undergone ERCP for biliary stenting because of biliary stenosis, cholecystitis, or bile leakage after surgery between January 2012 and July 2018 at Okayama University Hospital. The first cannula used in the procedure was the PR220Q (0.025-inch guidewire compatible, Olympus Medical Systems, Tokyo, Japan) or PR234Q (0.035-inch guidewire compatible, Olympus Medical Systems). The first guidewire used in the procedure was the 0.025-inch guidewire VisiGlide2 (Olympus Medical Systems) or 0.035-inch guidewire Revowave (Piolax Medical, Kanagawa, Japan). A 0.025-inch or 0.035-inch Radifocus (Terumo, Tokyo, Japan) was used when it was difficult to reach the target biliary branches. The guidewire was exchanged for a rigid, stiff guidewire (THSF; Cook Medical, Tokyo, Japan) when it was difficult to lead a biliary stent through the stenosis.

**Figure 2:**
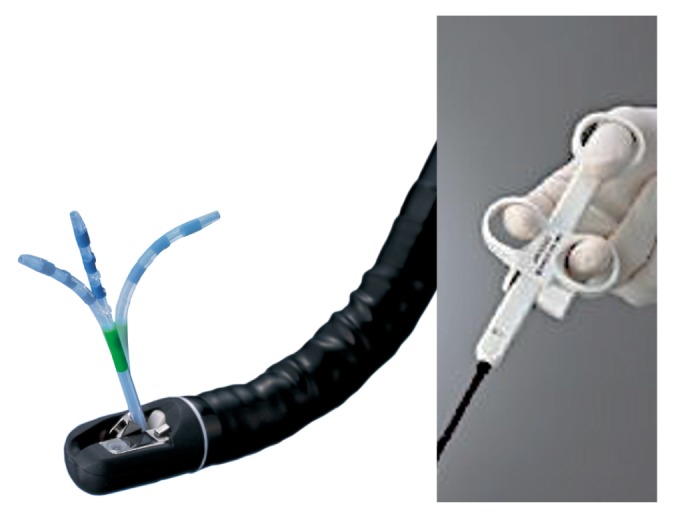
The Swing Tip has a flexible tip for manual control.

The Swing Tip is usually used for accessing the biliary branches if the procedure cannot be performed with any of these devices because of severe strictures or a crooked bile duct anatomy. Specifications and the image of the Swing Tip were shown in Table 1 and Figure 2. More specifically, there are two indications for using the Swing Tip: one is to advance a guidewire into a crooked bile duct; the other is to exchange the original guidewire for a rigid guidewire after successful guidewire placement (Video 1).

The indications for using the Swing Tip, technical success rate, clinical success rate, and adverse events were retrospectively evaluated. Technical success was defined as the successful placement of biliary stent into the target biliary branch. Clinical success was defined as the successful control of jaundice (50% decrease in total bilirubin within a week after the procedure), bile leakage, cholangitis, or cholecystitis.

## 3. Results

A total of 99 patients underwent ERCP using a Swing Tip. The characteristics of these patients are shown in Table 2. In all cases, 0.025-inch guidewires were used for initial cannulations. A total of 49 patients underwent ERCP using the Swing Tip to access the biliary branch, and 50 patients underwent a procedure to exchange a guidewire for a rigid guidewire (Video 1). In the former group, the conditions for which ERCP using the Swing Tip was performed were as follows: gallbladder drainage, n=17; benign biliary stricture, n=8; malignant biliary stricture, n=18; bile leakage after surgery, n=6. In the latter group, the procedures were as follows: gallbladder drainage, n=1; benign stricture, n=19; malignant biliary stricture, n=27; and bile leakage after surgery, n=3 (Table 3). The technical success rates in the former and latter groups were 44.9% (22/49) and 90% (45/50), respectively. The technical success rates of accessing the biliary branch at specific locations were as follows: gallbladder, 47% (8/17); B2, 80% (4/5); B4, 0% (0/1); B5, 42.8% (3/7); B6, 23% (3/13); B8, 75% (3/4). The technical success rate of exchanging guidewires at specific locations were as follows: gallbladder, 100% (1/1); B2, 100% (2/2); B3, 70% (7/10); B4, 100% (1/1); B5, 100% (5/5); B6, 92.6% (25/27); B7, 100% (1/1); B8, 100% (2/2); Bp, 100% (1/1) (Table 4). In all technically successful cases, clinical success was also achieved. No adverse events occurred in association with the use of the Swing Tip.

The 27 patients who failed to access the biliary branch were treated with other drainage approaches. Eleven patients (five with bile leakages, three with cholecystitis, and three with malignant stenosis) required percutaneous transhepatic biliary drainage (PTBD), and six patients with cholecystitis required surgical treatment. The other ten patients were able to be controlled with drainage of another branch. All patients ultimately achieved clinical success.

The five patients in whom a guidewire could not be exchanged underwent biliary stenting into alternative bile ducts. All of them ultimately achieved clinical success in terms of results.

## 4. Discussion

There are various technical challenges associated with ERCP aside from papillary cannulation. For example, selective seeking of biliary branches is technically challenging. A thin and flexible guidewire, such as a 0.025-inch guidewire, is most effective for the selection of biliary branches. However, a rigid guidewire is required to lead devices through severe stenoses or crooked ducts. Recently, a guidewire characterized by both flexibility and rigidity was developed [7], but sometimes exchange from a 0.025-inch to a 0.035-inch guidewire is required to lead certain devices. Severe stenoses can interfere with the passage of these devices, but other issues can also make leading devices difficult. For example, leading devices into bending bile ducts, such as cystic ducts or posterior branches, is sometimes challenging (Figure 1).

Endoscopic gallbladder drainage has been reported as an effective and safe treatment alternative to percutaneous treatment [8, 9]. It is technically challenging because identifying cystic ducts and leading devices into the gallbladder are often difficult. In these cases, we usually select a rigid guidewire such as the THSF, but the THSF is not flexible and not suitable for identifying branches. We therefore first use a flexible guidewire to identify the branches, and then a catheter that can be loaded with a THSF was used. This catheter leading technique is difficult, as is shown in Figure 1.

The Swing Tip was developed as a cannula with a manually controllable tip (Figure 2). This mechanism is instrumental for leading devices along a winding guidewire. The Swing Tip can be loaded onto a 0.035-inch guidewire including a THSF, thereby enabling secure stent placement.

In this study, 67 patients had stents successfully placed using a Swing Tip after technical failure with other devices. The Swing Tip was effective for identifying biliary branches and leading catheters through strictures. Accessing crooked ducts is sometimes difficult. The Swing Tip enables safe guidewire insertion along the course of the bile duct. 22 of the 49 (44.1%) patients in whom accessing a crooked bile duct with other devices failed were successfully treated for biliary drainage using the Swing Tip. Additionally, the Swing Tip was of particular value in exchanging guidewires for rigid ones. A rigid guidewire is sometimes required to lead devices through crooked biliary strictures; however, exchanging such guidewires is sometimes difficult because of the occurrence of crooked biliary stricture (Figure 1). As shown in Table 3, in 45 of the 50 (90%) cases in which exchanging a guidewire for another device failed, technical success was ultimately achieved using the Swing Tip. The Swing Tip is a relatively thick cannula but is superior for managing breakthrough strictures, as it has a controllable, flexible tip. Adjusting the angle of the tip with a handle affords advancing force. We recommend exchanging guidewires for rigid ones, such as a THSF, in cases with severe stricture, even if the Swing Tip can pierce more deeply and clearly dilate the stricture, because there are various factors of failures in device deliveries. Anatomical factors, such as crooked bile ducts, influence the ability to push a device, so a rigid guidewire is required for a successful procedure.

Seventeen of the 32 patients who failed to achieve technical success with other devices required percutaneous or a surgical approach. As shown in Table 3, accessing the biliary branch is difficult even if the Swing Tip is used, especially in crooked bile ducts such as the B6 bile duct because the Swing Tip has a limited range of motion. Additionally, the Swing Tip is rigid cannula because it is compatible with a 0.035-inch guidewire. This caused some failures in leading catheters due to the occurrence of severely crooked biliary stricture.

## 5. Conclusion

In conclusion, the Swing Tip was useful for ERCP, especially in patients who required guidewire exchange from 0.025 inches to 0.035 inches because of severe stenosis or a crooked bile duct. Endoscopic biliary drainage is a feasible and safe approach for treating biliary stricture, but there were some difficult cases in which technical success could not be achieved, and percutaneous or surgical treatment was ultimately required. The Swing Tip may enable such invasive treatments to be avoided; however, the present study is associated with some limitations due to its retrospective design and the small number of patients. Prospective studies are therefore required before we can draw any definitive conclusions.

## Figures and Tables

**Table 1:** Specifications of the Swing Tip.

Tip design	Platinum radiopaque tapered tip
Outer diameter of tube	2.95 mm
Outer diameter of distal end	4 Fr
Maximum bending angulation	Pull 90 degrees, push 30 degrees
Compatible guide wire	0.035 inch
Minimum scope channel diameter	3.2 mm

**Table 2:** Patient characteristics.

No. patients	99

Age (years) [median (range)]	66 (18-88)
Sex (M:F)	60:39
Disease	
cholangiocarcinoma	23
gallbladder carcinoma	8
pancreatic cancer	9
hepatocellular carcinoma	7
other cancer	5
cholecystitis	12
intrahepatic stone	4
bile leak	10
benign stricture	6
stenosis after surgery	15
Indication	
Access to biliary branch	49
Guidewire exchange	50

**Table 3:** Indications for the Swing Tip.

	Access to biliary branch (n=49)	Guidewire exchange (n=50)
gallbladder drainage	17	1
benign biliary stricture	8	19
malignant biliary stricture	18	27
bile leak after surgery	6	3

**Table 4:** The success rate according to the targeted biliary branch.

	Access to biliary branch	Guidewire exchange
	% (n)	% (n)
gallbladder	47 (8/17)	100 (1/1)
B2	80 (4/5)	100 (2/2)
B3	50 (1/2)	70 (7/10)
B4	0 (0/1)	100 (1/1)
B5	42.8 (3/7)	100 (5/5)
B6	23 (3/13)	92.6 (25/27)
B7	-	100 (1/1)
B8	75 (3/4)	100 (2/2)
Bp	-	100 (1/1)

Total	44.1 (22/49)	90 (45/50)

## Data Availability

The datasets used and/or analyzed during the current study are available from the corresponding author upon reasonable request.

## Abbreviations

ERCP: Endoscopic retrograde cholangiopancreatography
PTBD: Percutaneous transhepatic biliary drainage.

## Supplementary Materials

Supplementary Materials
** Video 1:** a video demonstrating the efficacy of the Swing Tip in leading devices into a crooked bile duct and exchanging a guidewire.
